# BCG Revaccination Does Not Protect Against Leprosy in the Brazilian Amazon: A Cluster Randomised Trial

**DOI:** 10.1371/journal.pntd.0000167

**Published:** 2008-02-13

**Authors:** Sérgio S. Cunha, Neal Alexander, Mauricio L. Barreto, Emilia S. Pereira, Inês Dourado, Maria de Fátima Maroja, Yury Ichihara, Silvana Brito, Susan Pereira, Laura C. Rodrigues

**Affiliations:** 1 Instituto de Saúde Coletiva, Universidade Federal da Bahia, State of Bahia, Brazil; 2 London School of Hygiene and Tropical Medicine, United Kingdom; 3 Fundação Alfredo da Matta, Manaus, State of Amazonas, Brazil; 4 Fundação Nacional de Saúde, State of Bahia, Brazil; World Health Organization, Switzerland

## Abstract

**Background:**

Although BCG has been found to impart protection against leprosy in many populations, the utility of repeat or booster BCG vaccinations is still unclear. When a policy of giving a second BCG dose to school children in Brazil was introduced, a trial was conducted to assess its impact against tuberculosis, and a leprosy component was then undertaken in parallel. Objective: to estimate the protection against leprosy imparted by a second dose of BCG given to schoolchildren.

**Methods and Findings:**

This is a cluster randomised community trial, with 6 years and 8 months of follow-up. Study site: City of Manaus, Amazon region, a leprosy-endemic area in Brazil. Participants: 99,770 school children with neonatal BCG (aged 7–14 years at baseline), of whom 42,662 were in the intervention arm (revaccination). Intervention: BCG given by intradermal injection. Main outcome: Leprosy (all clinical forms). Results: The incidence rate ratio of leprosy in the intervention over the control arm within the follow-up, in schoolchildren with neonatal BCG, controlled for potential confounders and adjusted for clustering, was 0.99 (95% confidence interval: 0.68 to 1.45).

**Conclusions/Significance:**

There was no evidence of protection conferred by the second dose of BCG vaccination in school children against leprosy during the trial follow-up. These results point to a need to consider the effectiveness of the current policy of BCG vaccination of contacts of leprosy cases in Brazilian Amazon region.

## Author Summary

BCG is a vaccine developed and used to protect against tuberculosis, but it can also protect against leprosy. In Brazil, children receive BCG at birth, and since 1996 a trial has been conducted to find out if a second dose of BCG administered to schoolchildren gives additional protection against tuberculosis. We use this trial to find out if such vaccination protects against leprosy. The trial was conducted in the Brazilian Amazon, involving almost 100,000 children aged 7–14 years who had received neonatal BCG. Half of them received a second dose of BCG at school, and the other half did not. We followed the children for 6 years and observed that there were as many new cases of leprosy in the vaccinated children as in the unvaccinated children. Therefore, we concluded that a second dose of BCG given at school age in the Brazilian Amazon offers no additional protection against leprosy.

## Introduction

BCG vaccination is given routinely to neonates to prevent tuberculosis in Brazil and in most of the world. BCG also protects against leprosy, with estimates of protection ranging from 20% to 90% [1],[2]. In Brazil, in addition to routine BCG vaccination at birth to prevent tuberculosis, BCG is officially recommended for household contacts of leprosy cases. In 1994 the Brazilian Ministry of Health expanded its tuberculosis control policy to recommend the routine BCG vaccination of school age children (around 7–14 years old). Given the high coverage of neonatal vaccination, this was effectively revaccination for most children. A large cluster randomised trial (BCG-REVAC) was started in 1996 to assess the effectiveness against tuberculosis of BCG vaccination of schoolchildren [3],[4]. One of the trial sites was the city of Manaus, which is also endemic for leprosy. In this city the trial objective was then expanded to estimate the effectiveness on leprosy. This paper reports the results of the BCG-REVAC trial in preventing leprosy based on follow-up from January 1999 to August 2006.

## Methods

Details of the methodology of the BCG-REVAC trial and of the leprosy component have been published elsewhere (regarding trial co-ordination, screening to detect leprosy cases before the trial, and sample size) [4],[5]. A CONSORT checklist is available in Text S1. We summarise here relevant methodological aspects.

The main objective of the leprosy component of the BCG-REVAC trial was to estimate the protection against all forms of leprosy given by one dose of BCG under routine conditions to schoolchildren aged 7–14 years in a population with coverage of neonatal BCG of about 89%. Our original hypothesis was that BCG revaccination would cause 50% reduction in incidence, based on the estimate observed in the trial in Malawi [6]. This is a vaccine effectiveness, pragmatic trial [7], rather than an efficacy trial. The study design attempted to reproduce the routine implementation of the policy of BCG vaccination of schoolchildren according to the 1994 recommendation.

The study site was the city of Manaus, in Amazonas State of Brazil, with about 1,500,000 inhabitants in 2002. The city is divided into 56 administrative districts, which in turn are grouped into 6 geographical areas (North, East, South, West, Centre West and Centre South). The new case detection rate (NCDR) has been around 6.5 cases per 10,000 per year of leprosy since the 1990s. In 1997 the NCDR was 6.6 (814 cases) in the total population and 4.9 (110 cases) in children aged 7–14 years. The trial study population was schoolchildren residing in the city, aged 7–14 years and attending state schools at the time of the trial implementation in 1998 (year of birth between 1984 and 1991). No child was excluded on the basis of previous history of tuberculosis or leprosy, mirroring the official recommendation for BCG vaccination to schoolchildren.

Randomisation. There are several reasons to do randomisation at cluster level in studies on infectious diseases [8]. In this trial, the main reason was operational: a list of schools with approximate numbers of students, but not names, was available. Without a list of student's names, individual randomization would be much harder given the very large number of children involved. The decision also considered the following advantages of cluster randomisation in this case: intervention (vaccination) more likely to be acceptable, as all schoolchildren within the same school would be allocated to receive or not to receive vaccination; simpler execution; large number of randomisation units (schools) expected to result in similar comparable allocation groups. Furthermore, since the recommendation was to vaccinate schoolchildren, schools represent the settings where this intervention would naturally be implemented in a real campaign.

There was no previous study on leprosy in which intra-class correlation (ICC) had been estimated, and ICC estimated by this study before the trial follow-up resulted in a negative value [5], which was interpreted as suggestive of no effect of clustering [9]. Therefore, although this was a cluster randomised trial, the initial sample size estimation made no allowance for clustering, and it was estimated 50,000 children in each allocation arm [5], using formulae in chapter 7 of Friedman et al. [10].

The randomisation followed several steps and was conducted using a list of schools provided by the local education department, with estimates of the number of schoolchildren in each school. Only schools with more than 50 schoolchildren in the target age group and in the main urban area of Manaus were included. First, the 56 districts were classified into strata according to the incidence of leprosy and tuberculosis in each district before the trial (1996). If the leprosy incidence (NCDR) in 1996 in a district was above the rate of the city as a whole, then it was categorised as “above” = 1, otherwise (below the city rate) as “below” = 0. The same procedure was used for tuberculosis, “above” or “below” the city rate. The 56 districts were thus grouped into 5 strata of incidence of leprosy and tuberculosis: four combinations of rate of tuberculosis and leprosy (above/above, above/below, below/above and below/below the rate of the city, respectively), and a fifth category with the districts with unavailable data on leprosy and tuberculosis. Second, the schools in the list were sorted by a) greater geographical areas, b) the 5 strata and then c) on the estimate of the number of schoolchildren at the target age group. Third, within the same geographical areas and 5 strata, the schools with the closest number of schoolchildren were then taken as a pair. Fourth, random numbers were generated by computer for each school, and in each pair the school with the smallest random number was allocated to the control arm, and the other was allocated to the intervention arm. When a school had no pair (odd number of schools in the stratum by geographical area and category on leprosy/tuberculosis), it was allocated at random (odd number was control, even number was intervention). The randomisation process was implemented by two researchers, S.S.C and S.P.

Three hundred and forty five (345) schools from the original list were used in the randomisation, selected based on number of students and being located in urban area. The number of schoolchildren was approximately estimated to be 161,736. However, subsequent field work showed that some of the information in the list was inaccurate, and schools in the list were excluded for several reasons: number of schoolchildren smaller than 50; school closed during the time of trial implementation; school mostly included children with special needs. Also, a single school was listed as two schools because it was based over two sites. Finally, from the original 345 schools, only 286 were eventually entered in the trial.

### Recruitment

After randomisation, visits to these 286 schools were conducted between July and October 1998 to collect children's data, including BCG scar reading and BCG vaccination. Data were transcribed from school records, and children were examined to identify those who had received neonatal BCG vaccination, based on BCG scar.

### Intervention

Children in the schools allocated to vaccination received 0.1 ml of lyophilised BCG produced in Brazil, Moreau strain, administered by intradermal injection [4]. Four different batches were used but vaccination in any one school was done with a single batch. Vaccination began in September and finished in December 1998. Children in the control arm did not receive a placebo.

**Figure 1:**
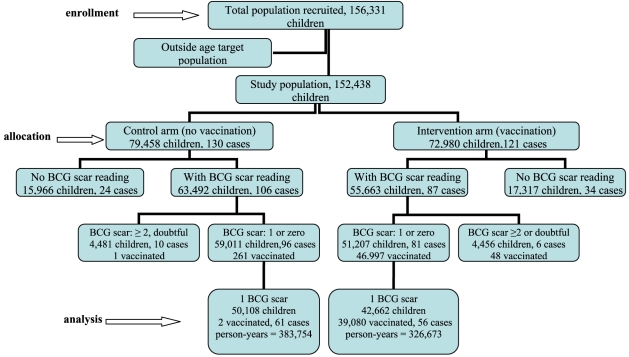
Flow of clusters and individual participants through each stage.

Data were collected from 156,331 schoolchildren (nearly 70% of the estimated population of Manaus aged 7–14 years in 1998), and 3,893 children were later excluded because they were outside this age range (see Figure 1). The analysis plan originally proposed to estimate the vaccine effect in children with either no or one BCG scar as observed at baseline, consisting of 110,218 children: 51,207 in the intervention arm (allocated to vaccination) of whom 46,997 were vaccinated (92%). Reasons for not vaccinating the remaining 4,210 children included refusals, withdrawals, and changes in school [5].

### Follow-up

Because most leprosy in Manaus is tuberculoid, which has a shorter incubation period [11], and the protection of BCG against leprosy was observed very soon (after 1 year) in some trials [12],[13], the decision was made to start the follow-up from 1^st^ January 1999 (and to end in August 2006, see analysis). First January 1999 corresponded to more than 2 months after vaccination for nearly 50% of the vaccinated children (range 22–100 days).

Allocation concealment refers to the process of making the investigators not able to know the randomisation sequence between the time it is generated and the time a particular code is allocated to study unit [14]. In our trial, the allocation was done immediately after the sequence generation, so the allocation was in effect concealed.

Most cases were diagnosed at the leprosy reference centre in Manaus, where the local leprosy control programme and its surveillance system are located. In the study site, would be very unusual for a suspected case of leprosy to be identified at school and referred to the health services for diagnosis and treatment. Leprosy cases normally go spontaneously to routine health services, or are referred from primary services to the leprosy reference centre, as several services have dermatologic clinics. Therefore, detection rates are not expected to differ systematically between control and intervention schools.

In order to give blind diagnosis at the reference centre, most patients (85 out of 91) up to 2001 had their right deltoid area covered by an adhesive tape on entering the medical offices for investigation of leprosy the diagnosis, however, after 2001 this procedure was found to be little used and was then definitely discontinued. Physicians were also continually asked to refrain from inquiring about the BCG status until a definitive diagnosis of leprosy was made, unless the physician has judged it was necessary to know the BCG status for good clinical practice. Therefore, the trial can not be considered as being blind, and furthermore the absence of placebo meant that neither those administering the interventions, nor the participants were blind to their assignment.

### Case diagnosis and classification

Cases were thus classified into multibacillary (MB) and paucibacillary (PB) as reported in medical records and histopathology exams, and diagnosis made following the routine procedures used in the reference service, based mostly on combination of clinical signs, baciloscopy and histopathology and on WHO criteria [15]. Bacillary index and biopsies are routine procedures for all suspect cases, unless there are contraindications such as in young children, facial lesions, and refusal by the patients. Data on anaesthesia, aspects of skin lesions and thickening nerve are routinely collected during the clinical examination. Such data on the diagnosis and classification were thus periodically retrieved as described in the medical records soon after the diagnosis, but any doubt on classification and diagnosis was discussed with the professionals responsible by the patient or exam. Cases are routinely classified according to grade of disability (0 = no anaesthesia or deformity in hands or feet, and no eye problem; grade 1 = with anaesthesia but no deformity, or with eye problem but vision not severely affected; grade 2 = visible deformity or vision severely affected [16]), and this was information was also retrieved. The difficulty of diagnosing leprosy is well known, and one way to overcome such difficulties is to categorise the leprosy cases in “certainty levels” based on typical signs [17]. There was an initial attempt during the first/second year of follow-up to register relevant signs and symptoms into a standardised questionnaire to be completed by the doctors responsible by the assistance of each suspect case, as well as histopathology exams should follow specific procedures and findings annotated into a standardised form. However, the completion rate was low and the information from these forms was not used. There was no independent review panel for deciding on the final diagnosis of leprosy cases. Given that it is an effectiveness (pragmatic) trial, which means it was aimed at assessing vaccine effect under routine conditions, it was decided that all leprosy cases reported in the surveillance system should be included in the analysis, but that the analysis should include a comparison of cases classified into 2 levels of certainty based on laboratory and clinical presentation: those with confirmed histopathology, or positive baciloscopy or thickening nerve, versus those without any of these data. Therefore, we performed sub-group analysis for these two levels.

Leprosy cases detected by the local surveillance system were linked to the trial population by matching information from the notification to records in the trial data-base (date of birth, name of case and name of the case's mother) [5]. There were 650 leprosy cases detected by the local surveillance from 1999 to August 2006 in the target age population and residing in Manaus, of whom 253 cases were identified in the 156,331 total trial population, and 117 cases (out of 253) were among those 92,770 children with one BCG scar (see Figure 1). In this group, 91.6% of children in intervention arm (n = 42,662) actually received BCG in the trial, and only 2 children in the control arm (n = 50,108) were wrongly vaccinated (originally enrolled in school in the control arm but actually attending school in intervention arm).

Surveillance for adverse events. The routine passive surveillance of adverse events was enhanced. A letter containing information on BCG adverse events was distributed to all children on the day of vaccination to motivate parents to take their children to a health facility if they had a health problem following vaccination. Teachers in the trial schools and health workers in the reference medical centres for tuberculosis and leprosy were made aware of the trial and alerted to possible BCG adverse events. Suspect adverse events were diagnosed in the health facilities and treatment provided. This vaccine safety surveillance continued for 4 months after the end of vaccination.

### Ethics

The BCG-REVAC trial received ethical approval by the Brazilian National Ethical Committee (CONEP, *Comissão Nacional de Ética em Pesquisa*) [5]. The trial is registered with an International Standard Randomised Controlled Trial Number, ISRCTN07601391 (http://www.controlled-trials.com/ISRCTN07601391).

### Analysis

The main outcome of the leprosy component of this trial consisted of leprosy cases diagnosed in the health facilities in Manaus, expressed as the NCDR of leprosy per 10,000 person years in the two arms during the trial follow-up. Baseline characteristics of the population are presented at individual and cluster levels separately for the intervention and control arms, and for those excluded and those included in the vaccine effect estimation. No significance test was used to assess differences on baseline characteristics [18]. All calculations were based on rates and rate ratios estimated by Poisson regression. The 95% confidence intervals were based on robust (“sandwich”) variance estimator, specifying school (not pair of schools) as cluster to allow for clustering within schools [19]. Rates and rate ratios were also adjusted for covariates to correct for any imbalance between intervention groups, and covariate adjustments are stated below when describing each analysis.

BCG vaccine protection was estimated as (1-RR)×100, RR being the ratio of the rate in the intervention arm over the rate in the control arm, among those 92,770 individuals with one BCG scar, with intention-to-treat analysis. Statistical analysis was done in STATA version 7.0.

### Departure from protocol

Interim analysis of the leprosy results was not planned. Originally, the analysis was planned for all leprosy cases involving those with no or one BCG scar, which was planned to have a power of 80% to detect a vaccine protection of 50%. This analysis was conducted in 2003, but not published [20]. However, it was subsequently recognised that the most important estimate would be for those with neonatal BCG, rather than for all children regardless of previous BCG status, because Brazil currently achieves a high neonatal BCG coverage rate, which means that, in the near future, most individuals will have received neonatal BCG. In addition, there was not enough power to assess heterogeneity of vaccine effect according to one or zero BCG scar. The decision was therefore made to redo the analysis, among only those children with one BCG scar at entry, at a time when a study power of 80% was projected to have been achieved for this sub-population. Based on the hypothesised rate ratio of 0.5, and 91% coverage in the vaccine arm, this was achieved with the 117 cases detected up to August 2006 [21]. Hence the decision was made to analyse the cases accumulated up to this time. This paper therefore reports the estimate for vaccine protection among children with one BCG scar, that is, effect of re-vaccination. At the moment of this analysis, the number of cases among those with no BCG scar was not sufficient for a study power of 80% and thus vaccine protection was not estimated in this group.

## Results

The number of leprosy cases detected in the trial and number of children are shown in Figure 1. The baseline characteristics of the two allocation arms were similar regarding gender, age at entry into the trial (Table 1). A higher proportion of children in intervention arm were in schools located in areas that had higher incidence of tuberculosis and leprosy (NCDR) before the trial (bold numbers in Table 1).

**Table 1:** Comparability of the baseline characteristics of the trial population, according to BCG scar.

Baseline Characteristics	Schoolchildren without BCG Scar Reading, without BCG Scar or >1 Scar (Excluded in the Analysis of Vaccine Effect)		Schoolchildren with 1 BCG Scar (Included in the Analysis of Vaccine Effect)	
	Control	Intervention	Control	Intervention
Total number	29,350	30,318	50,108	42,662
Median number per school (percentiles 25%–75%)	181 (113–271)	193 (121–265)	302 (209–433)	274 (159–383)
Number of males (%)	14,572 (49.7)	15,232 (50.2)	24,530 (49.0)	20,880 (48.9)
Average percentage of males per school	50.0%	50.0%	48.9%	49.2%
Mean age in years at entry into the trial (SD^a^)	11.5 (2.2)	11.3 (2.2)	11.34 (2.11)	11.17 (2.05)
Mean age in years at entry into the trial, per school (SD^a^)	11.2 (1.3)	11.0 (1.1)	11.2 (1.3)	11.1 (1.2)
Rates of tuberculosis in 1996^b^ and number of schoolchildren (%)				
14.5 to 125 per 10,000	20,152 (68.7)	19,186 (63.3)	35,436 (70.7)	27,819 (65.2)
131.6 to 618 per 10,000	8,390 (28.6)	11,132 (36.7)	13,875 (27.7)	14,843 (34.8)
Areas without data on tuberculosis rates	808 (2.8)	0	797 (1.6)	0
Rates of leprosy in 1996^c^ and number of schoolchildren (%)				
0 to 4.57 per 10,000	6,539 (22.3)	5,878 (19.4)	11,334 (22.6)	6,924 (16.2)
4.83 to 6.38 per 10,000	7,545 (25.7)	6,967 (23.0)	12,636 (25.2)	9,460 (22.2)
6.52 to 7.33 per 10,000	6,958 (23.7)	5,259 (17.4)	12,215 (24.4)	9,221 (21.6)
7.82 to 10.92 per 10,000	3,572 (12.2)	5,139 (17.0)	6,449 (12.9)	6,685 (15.7)
12.21 to 66.94 per 10,000	4,234 (14.4)	7,075 (23.3)	6,791 (13.6)	10,372 (24.3)
Areas without data on leprosy rates	502 (1.7)	0	683 (1.4)	0

a SD is standard deviation.

b Rates of tuberculosis before the trial and geographical areas where schools were located, the two categories correspond to the rate below and above of the city.

c Rates of leprosy before the trial and geographical areas where schools were located.

Among those excluded from the analysis for having no BCG scar reading, or with no scar or >1 scar, the leprosy rates (per 10,000) were 3.07 (95% C.I.: 2.43 to 3.89; 69 cases in 224,605 person years) in the control arm, and 2.80 (95% C.I.: 2.20 to 3.57; 65 cases in 232,016 person years) in the intervention arm. All results below are restricted to those with one BCG scar.

Description of cases. Among the 117 cases in the population with 1 prior BCG scar, 30 cases were MB and 87 PB. Most cases (112) had grade 0 (no disability); in the intervention arm there were 3 cases with grade 1 and 2 cases with grade 2. Nineteen cases had nerve thickening (16.2%): 8 (13.1%) in control arm, and 11 (19.6%) in intervention. Fifty seven cases (48.7%) were confirmed by histopathology: 29 (47.5%) in control arm, and 28 (50.0%) in intervention. The mean age at diagnosis was 15.6 years (sd 3.1) in control and 14.2 (sd 3.0) in intervention. There were 47.5% of male cases in the control arm (29/61) and 51.8% (29/56) in the intervention arm.

The rates (per 10,000 person-years) of MB cases were 0.36 in the control arm (14 cases/383,754 person years) and 0.49 in the intervention arm (16/326,673 person years). For PB the rate was 1.22 in both arms. The rates by calendar year separately and allocation arm are shown in Table 2. There was a borderline statistically significant increase in the rate in the intervention arm in the first year of follow-up (1999), the rate ratio being 2.50 (95% C.I.: 0.99 to 6.35) (Table 2). This increase in the first year was observed for PB and MB cases: the rate ratio was 2.63 (95% C.I.: 0.31 to 22.00) for MB cases and 2.91 (95% C.I.: 0.87 to 7.20) for PB cases, adjusted as in Table 2.

**Table 2:** Rates of leprosy separately for calendar year during the follow-up period among those with 1 BCG scar.

Allocation Groups	Year of Follow-Up							
	1999	2000	2001	2002	2003	2004	2005/6^a^	Total
**Intervention**								
Cases	14	10	5	4	8	6	9	56
Person years	42,653	42,643	42,363	42,631	42,625	42,617	70,868	326,673
Rate × 10,000	3.28	2.35	1.18	0.94	1.88	1.41	1.27	1.71
**Control**								
Cases	7	9	11	4	9	5	16	61
Person years	50,105	50,096	50,086	50,078	50,073	50,065	83,250	383,754
Rate × 10,000	1.40	1.80	2.20	0.80	1.80	1.00	1.92	1.59
**Rate ratio** b	2.50	1.21	0.41	0.85	1.02	1.18	0.71	0.99
95% C.I.	0.99 to 6.35	0.49 to 3.00	0.13 to 1.32	0.17 to 4.11	0.36 to 2.88	0.33 to 4.28	0.33 to 1.55	0.69 to 1.43

a Until August 2006.

b Based on Poisson regression with robust variance estimator specifying school as cluster, and controlled for incidence of tuberculosis and NCDR of leprosy in geographical areas before the trial (all as categorical variables as in Table 1), sex, and year of birth. All estimates excluding 870 school children (being 2 leprosy cases in control group, 1 in 1999 and other in 2005–6, both PB cases) who had no data for tuberculosis and/or leprosy in geographical area.

The rate ratio between allocation arms is shown in Table 3, separately for MB and PB, controlled for study variables and adjusted for effect of clustering. There was no evidence of protection by the second dose of BCG during the follow-up period. For the whole study period, the robust standard error of the intervention-over-control rate ratios (controlled for the variables as in Table 3) arms were 0.18987 adjusted for clustering and 0.18581 not adjusted for clustering. We estimated design effect as (0.18987/0.18581)^2^ = 1.0442. Given that the design effect is 1+(n–1)×ICC, where ICC is the intra-class correlation coefficient and n is the mean cluster size ( = 327), the ICC was estimated as 0.00013568.

**Table 3:** Rate ratios between rate in intervention arm over rate in control arm, among those with 1 BCG scar, whether controlled for study variables, separately for clinical forms.

Clinical Forms and Follow-Up Period	Allocation Group		Rate Ratio^a^ (95% C. I.)	
	Intervention	Control	Leprosy rate in 1996 Used as:	
			5 Categories	Continuous
**For the whole follow-up period**	Py^b^ ** = **326,673; cases = 56	Py^b^ = 377,095; cases = 59	—	
**Total cases n = 115**				
Rate ratio, crude estimate adjusted for clustering^c^	—	—	1.10(0.73 to 1.64)	
Rate ratio, adjusted for confounders but not for clustering^d^	—	—	0.99(0.69 to 1.43) 1.07(0.74 to 1.54)	
Rate ratio, adjusted for confounders and for clustering^e^	—	—	0.99(0.68 to 1.45) 1.07(0.72 to 1.58)	
**Multibacillary cases n = 30**	16	14	—	
Rate ratio, crude estimate adjusted for clustering^c^	—	—	1.32(0.67 to 2.61)	
Rate ratio, adjusted for confounders but not for clustering^d^	—	—	1.39(0.69 to 2.79) 1.33(0.66 to 2.69)	
Rate ratio, adjusted for confounders and for clustering^e^	—	—	1.39(0.72 to 2.66) 1.33(0.69 to 2.59)	
**Paucibacillary cases n = 85**	45	40	—	
Rate ratio, crude estimate adjusted for clustering^c^	—	—	1.00(0.61 to 1.64)	
Rate ratio, adjusted for confounders but not for clustering^d^	—	—	0.86(0.56 to 1.33) 0.98(0.64 to 1.51)	
Rate ratio, adjusted for confounders and for clustering^e^	—	—	0.86(0.54 to 1.37) 0.98(0.60 to 1.62)	
**Excluding the first year of follow-up**	Py^b^ = 284,019; cases = 42	Py^b^ = 333,649; cases = 53	—	
**Total cases n = 95**				
Rate ratio, crude estimate adjusted for clustering^c^	—	—	0.91(0.59 to 1.43)	
Rate ratio, adjusted for confounders but not for clustering^d^	—	—	0.83(0.55 to 1.24) 0.91(0.61 to 1.37)	
Rate ratio, adjusted for confounders and for clustering^e^	—	—	0.83(0.55 to 1.25) 0.91(0.59 to 1.41)	
**Multibacillary cases n = 26**	13	13	—	
Rate ratio, crude estimate adjusted for clustering^c^	—	—	1.15(0.54 to 2.49)	
Rate ratio, adjusted for confounders but not for clustering^d^	—	—	1.28(0.60 to 2.76) 1.30(0.60 to 2.79)	
Rate ratio, adjusted for confounders and for clustering^e^	—	—	1.28(0.63 to 2.62) 1.30(0.63 to 2.65)	
**Paucibacillary cases n = 69**	29	40	—	
Rate ratio, crude estimate adjusted for clustering^c^	—	—	0.84(0.52 to 1.35)	
Rate ratio, adjusted for confounders but not for clustering^d^	—	—	0.68(0.42 to 1.10) 0.80(0.49 to 1.30)	
Rate ratio, adjusted for confounders and for clustering^e^	—	—	0.68(0.40 to 1.16) 0.80(0.45 to 1.43)	

a All estimates excluding 870 school children (being 2 leprosy cases in control group, 1 in 1999 and other in 2005/6, both PB cases) who had no data for tuberculosis and/or leprosy in geographical area.

b Py: total person years.

c Based on Poisson regression with robust variance estimator specifying cluster (schools), without controlling for confounders.

d4 Based on Poisson regression with robust variance estimator without specifying cluster (not adjusted for clustering effect), and controlled for sex, year of birth, incidence of tuberculosis (categorical as in Table 1) and NCDR of leprosy (categorical as in table 1 or continuous variable) in geographical areas before the trial.

e The same to the regression above, but specifying schools as cluster (adjusted for clustering effect).

The rate ratio between those vaccinated and not vaccinated, regardless of allocation arm (using on-treatment analysis) for the whole trial follow-up was, for all leprosy cases 0.97 (95% C.I.: 0.67 to 1.41), for MB cases 1.08 (0.54 to 2.15) and PB cases 0.92 (0.59 to 1.43), after controlling for study variables as in Table 3. After excluding the first year of follow-up, the rate ratio for all leprosy cases was 0.81 (0.54 to 1.21), for MB cases was 1.09 (0.50 to 2.37), and for PB cases was 0.71 (0.43 to 1.15).

The rate ratio intervention over control arms, based on cases with confirmed histopathology, positive baciloscopy or thickening nerve (n = 60) was 1.51 (95% C.I.: 1.04 to 2.19), after control for sex, BCG scar, year of birth, and the previous rates of leprosy and tuberculosis in the districts where schools were located. For those with neither confirmed histopathology, positive baciloscopy nor thickening nerve, but based on clinical judgement on typical skin lesion and presence of anaesthesia (n = 57), it was 1.03 (95% C.I.: 0.76 to 1.39).

Among the total 152,438 individuals enrolled in the study, 47,307 individuals were vaccinated, and 18 cases were reported with adverse events related to BCG (risk of 3.80 per 10,000). Eight cases was due to ulcer greater than 1 centimetre, 7 cases had cold abscess, and the 3 remaining cases had axillary lymph node enlargement without suppuration, “hot” abscess with suppuration and nodule in vaccination site. Children without previous BCG scar had a risk of 3.67 (3 cases/8,176 individuals), and those with 1 BCG scar (revaccination) the risk was of 3.84 (15 cases/39,067), this corresponded to a risk ratio of 1.05 (95% C.I.: 0.31 to 3.53), after adjustment for effect of clustering, sex and year of birth.

## Discussion

This study found no evidence of protection of the second dose of BCG against all forms of leprosy among school children within 6 years and 8 months of follow-up. This remained after controlling for potential confounders and adjusting for effect of clustering. The confidence interval of 0.72 to 1.58 (for the all cases and the whole period, controlled for covariates and adjusted for clustering) is consistent with a vaccine protection of up to 28% and an increase in leprosy in those vaccinated up to 58%. We conclude that the results did not support the original hypothesis of a vaccine protection of 50%, and additional follow-up is thus not planned. There was no statistically significant vaccine protection against MB and PB cases, but this analysis was not powered to evaluate protection separately for clinical forms at this duration of follow up. We can speculate some alternative hypotheses why the expected protection by revaccination was not observed.

First, differential detection rate. The diagnosis of most leprosy cases was blind to vaccination status in the first two years, but after 2001 the procedures recommended for blind diagnosis were not used. However, diagnosis was based on routine procedures as before the trial, and it is unlikely that special attention was paid to patients' revaccination status: BCG history tends to not be considered in routine procedures in Manaus. It is also unlikely that patients themselves sought medical attention differentially according to whether they were vaccinated in the trial. So a subjective assessment would suggest that distortion of the estimates due to differential detection rate because of lack of blindness seems unlikely.

Second, the result could be due to misdiagnosis. The difficulty of diagnosing leprosy is well known [17]. If patients with other skin lesions were wrongly diagnosed as having leprosy and were included in the study, this would lead to an underestimation of the vaccine effect. However, the quality of diagnosis in the study was high: most leprosy cases had the typical signs or laboratory findings of leprosy. This is strengthened by the fact that this study was based on a reference centre for leprosy diagnosis and treatment, with very experienced clinicians; and protection did not increase with certainty of diagnosis. Therefore, it is unlikely that false positive diagnoses would be responsible for lack of vaccine effect.

Third, linkage of cases. The linkage was done blind to vaccination status, and so it is unlikely that any failure in linkage of cases would be differential. Fourth, this could be due to imbalances in the baseline characteristics in the comparison groups or selection bias when defining the study population. Indeed, the incidence of leprosy (NCDR) in the geographical areas where vaccinated and control schools were located were unbalanced, despite the randomisation process. This imbalance must have been caused from the inaccuracy of the list of schools used in the randomisation process and the exclusion after allocation. This unbalance could potentially distort the vaccine effect, which is why we controlled for sex, age, year of birth, and previous incidence of tuberculosis and leprosy in the analysis.

Fifth, poor vaccine administration and vaccine storage. The vaccination was done following routine procedures, however, temperature was regularly checked and no problems were detected. Furthermore, vaccine strain, the staff and procedures used in the trial were similar to used in neonatal vaccination, and neonatal BCG vaccination was shown to be protective against leprosy in the cohort study nested on the trial [22]. Therefore, vaccine administration and vaccine storage were at worst similar to those which have resulted in protection of 90% in the nested cohort study for neonatal vaccination.

Sixth, the lack of vaccine effect could not be due to a distortion caused by vaccination by the trial of individuals in the control arm, because very few individuals in the control arm among those with one BCG scar were vaccinated (n = 2 of 50,108). BCG vaccination in schoolchildren was suspended in the study site because of the trial during the study follow-up, and definitely in the whole country in 2006. Nevertheless, 312 schoolchildren in Manaus were reported as wrongly vaccinated in routine in 2002 although none belonged to the trial population. No other child in the target age group was reported vaccinated by public health services [23], and the number of children who received BCG in private services is negligible, given that it is offered free of charge by public services.

Eighth, the presence of HIV infectionin Manaus. There are no data on infection, but the annual average number of reported AIDS cases aged 15–19 years between 2001 and 2004 was 13 cases, with a estimated population in this age group of 182,745 in 2004, therefore HIV infection is expected to be very low and not have any effect on the vaccine effect observed [23].

The increase in the leprosy rate during the first year of follow-up in the intervention arm deserves comment. There is some prior evidence of BCG vaccination increasing the risk of leprosy in the initial follow up period, probably due to change in disease progression (“negative effect”) [24]–[26]. However, revaccination was also not protective when the first year of follow-up was excluded, and the increase in the first year of follow up was not responsible for the absence of effect observed for the whole period.

How does this result compare to previous studies? Among trials, in the study in Papua New Guinea the participants received different number of doses during the trial. However, the reports did not present vaccine protection or data separately for the number of doses received, although it was stated that the number of doses did not affect the vaccine protection [13]. In the last trial in India (1991), the study participants included those with and without previous vaccination, but separate data for previous vaccination were not shown, although it was reported that previous vaccination did not affect the protection conferred by the vaccine given by the trial [27]. In contrast, the trial in Malawi estimated the vaccine protection given by a second dose, and showed a statistically significant vaccine protection of 50% [6], but there were several different characteristics, including: different BCG strain (from Glaxo); screening to remove leprosy cases before vaccination; randomisation by individual rather than cluster; a mix of passive and active case detection; and broader age range from infants to adults. Among case-control studies, two assessed vaccine effect by number of doses and both showed additional protection with more than one dose [28],[29], but only in one study was a statistically significant trend of higher protection with increased number of doses [28]. A recent meta-analysis of BCG vaccination against leprosy concluded that additional doses offer additional protection [30]. This meta-analysis also found strong evidence of heterogeneity between studies. In our oppinion, although there is indeed some evidence for additional protection, the results are variable and do not support an unequivocal conclusion for additional protection by more than one dose in all sites.

Had the follow-up period been longer, would it be possible to observe vaccine protection in the years to come? There has been a recent report of BCG protection against tuberculosis lasting for at least 20 years in Brazil [31]. Indeed, in the last trial in India (1991) no vaccine protection was observed in the first years, but a statistically significant result was observed afterwards [24]. Therefore it is theoretically possible that continued follow up will demonstrate protection in coming years, but it is uncertain that this would be of public health importance.

BCG revaccination is currently recommended to contacts of leprosy patients in Brazil [32]. The results of this trial are not directly applicable to the setting of contacts, as protection of revaccination might be different given the close exposure in contacts, but we suggest that the effectiveness of revaccination in contacts must be evaluated to inform a review of such recommendation.

## Supporting Information

Text S1 CONSORT Checklist
(0.06 MB DOC)
