# Supplementary material for: BCG Revaccination Does Not Protect Against Leprosy in the Brazilian Amazon: A Cluster Randomised Trial
Source: PLoS Negl Trop Dis. 2008 Feb 13;2(2):e167. doi: 10.1371/journal.pntd.0000167 (PMC2238709; doi:10.1371/journal.pntd.0000167)
Supplement: Text S1 — CONSORT Checklist (0.06 MB DOC) [file pntd.0000167.s001.doc]

**CONSORT Checklist**

from:

Campbell MK, Elbourne DR, Altman DG. CONSORT statement: extension to cluster randomised trials. BMJ. 2004 Mar 20;328(7441):702-8.

| **Paper section and topic** | **Item** | **Descriptor** | **Page of manuscript** |
| --- | --- | --- | --- |
| **Title and abstract** |  |  |  |
| Design | 1 | How participants were allocated to interventions (eg random allocation, randomised, or randomly assigned), *specifying that allocation was based on clusters* | 1 and 2 |
| **Introduction** |  |  |  |
| Background | 2 | Scientific background and explanation of rationale, *including the rationale for using a cluster design* | 3 |
| **Methods** |  |  | 4-10 |
| Participants | 3 | Eligibility criteria for participants *and clusters* and the settings and locations where the data were collected | Participants: 4  Clusters: 5 |
| Interventions | 4 | Precise details of the interventions intended for each group, *whether they pertain to the individual level, the cluster level, or both,* and how and when they were actually administered | 6 |
| Objectives | 5 | Specific objectives and hypotheses *and whether they pertain to the individual level, the cluster level, or both* | 4 |
| Outcomes | 6 | Report clearly defined primary and secondary outcome measures, *whether they pertain to the individual level, the cluster level, or both*, and, when applicable, any methods used to enhance the quality of measurements (eg multiple observations, training of assessors) | 9 |
| Sample size | 7 | How *total* sample size was determined *(including method of calculation, number of clusters, cluster size, a coefficient of intracluster correlation (ICCor k), and an indication of its uncertainty*) and, when applicable, explanation of any interim analyses and stopping rules | 5 |

| Randomisation: |  |  |  |
| --- | --- | --- | --- |
| Sequence  generation | 8 | Method used to generate the random allocation sequence, including details of any restriction (eg blocking, stratification, *matching*) | 5 |
| Allocation  concealment | 9 | Method used to implement the random allocation sequence, *specifying that allocation was based on clusters rather than individuals and* clarifying  whether the sequence was concealed until interventions were assigned | 7 |
| Implementation | 10 | Who generated the allocation sequence, who enrolled participants, and who assigned participants to their groups | 6 |
| Blinding  (masking) | 11 | Whether participants, those administering the interventions, and those assessing the outcomes were blinded to group assignment. If done, how the success of blinding was evaluated | 7 |
| Statistical methods | 12 | Statistical methods used to compare groups for primary outcome(s) *indicating how clustering was taken into account*; methods for additional  analyses, such as subgroup analyses and adjusted analyses | 9 |
| **Results** |  |  |  |
| Participant flow | 13 | Flow of *clusters and* individual participants through each stage (a diagram is strongly recommended). Specifically, for each group report the numbers of *clusters and* participants randomly assigned, receiving intended treatment, completing the study protocol, and analysed for the primary outcome. Describe protocol deviations from study as planned, together with reasons | Flowchart  Protocol deviations: 9 |
| Recruitment | 14 | Dates defining the periods of recruitment and follow up | Recruitment: 6  Follow-up: 7 |
| Baseline data | 15 | Baseline information for each group *for the individual and cluster levels as*  *applicable* | Table 1 |
| Numbers analysed | 16 | Number of *clusters and* participants (denominator) in each group included in each analysis and whether the analysis was by intention to treat. State the  results in absolute numbers when feasible (eg 10/20 not 50%) | Flowchart |
| Outcomes and  estimation | 17 | For each primary and secondary outcome, a summary of results for each  group *for the individual or cluster level as applicable*, and the estimated  effect size and its precision (eg 95% confidence interval) *and a coefficient of*  *intracluster correlation (ICC or k) for each primary outcome.* | Results-Table 3  ICC: 12 |
| Ancillary analyses | 18 | Address multiplicity by reporting any other analyses performed, including subgroup analyses and adjusted analyses, indicating those prespecified and those exploratory | 10 |
| Adverse events | 19 | All important adverse events or side effects in each intervention group | 12 |
| **Discussion** |  |  |  |
| Interpretation | 20 | Interpretation of the results, taking into account study hypotheses, sources of potential bias or imprecision and the dangers associated with multiplicity  of analyses and outcomes. | 13-15 |
| Generalisability | 21 | Generalisability (external validity) *to individuals and/or clusters (as relevant)* of the trial findings | 16 |
| Overall evidence | 22 | General interpretation of the results in the context of current evidence | 15 |
